# CAPE: An R Package for Combined Analysis of Pleiotropy and Epistasis

**DOI:** 10.1371/journal.pcbi.1003270

**Published:** 2013-10-24

**Authors:** Anna L. Tyler, Wei Lu, Justin J. Hendrick, Vivek M. Philip, Gregory W. Carter

**Affiliations:** 1 The Jackson Laboratory, Bar Harbor, Maine, United States of America; 2 Department of Electrical and Computer Engineering, Duke University, Durham, North Carolina, United States of America; University of Canterbury, New Zealand

## Abstract

Contemporary genetic studies are revealing the genetic complexity of many traits in humans and model organisms. Two hallmarks of this complexity are epistasis, meaning gene-gene interaction, and pleiotropy, in which one gene affects multiple phenotypes. Understanding the genetic architecture of complex traits requires addressing these phenomena, but interpreting the biological significance of epistasis and pleiotropy is often difficult. While epistasis reveals dependencies between genetic variants, it is often unclear how the activity of one variant is specifically modifying the other. Epistasis found in one phenotypic context may disappear in another context, rendering the genetic interaction ambiguous. Pleiotropy can suggest either redundant phenotype measures or gene variants that affect multiple biological processes. Here we present an R package, R/cape, which addresses these interpretation ambiguities by implementing a novel method to generate predictive and interpretable genetic networks that influence quantitative phenotypes. R/cape integrates information from multiple related phenotypes to constrain models of epistasis, thereby enhancing the detection of interactions that simultaneously describe all phenotypes. The networks inferred by R/cape are readily interpretable in terms of directed influences that indicate suppressive and enhancing effects of individual genetic variants on other variants, which in turn account for the variance in quantitative traits. We demonstrate the utility of R/cape by analyzing a mouse backcross, thereby discovering novel epistatic interactions influencing phenotypes related to obesity and diabetes. R/cape is an easy-to-use, platform-independent R package and can be applied to data from both genetic screens and a variety of segregating populations including backcrosses, intercrosses, and natural populations. The package is freely available under the GPL-3 license at http://cran.r-project.org/web/packages/cape.

This is a *PLOS Computational Biology* Software Article

## Introduction

**Figure 1:**
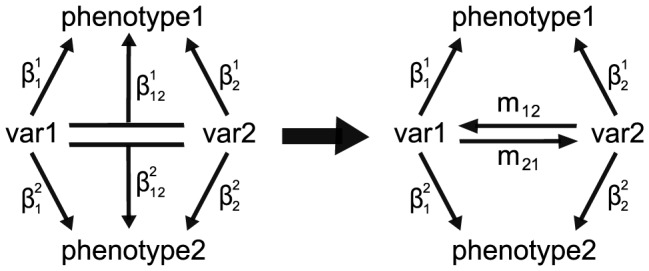
Overview of coefficient reparametrization for two phenotypes. On the left, main effect and interaction parameters for two variants (*var1* and *var2*) are derived from pairwise regressions (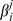). The interaction coefficients are reparametrized as 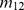 and 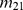 on the right, which describe variant-to-variant influences that fit both phenotypes via indirect associations. For 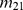 the source variant is *var1* and the target variant is *var2*, with the source and target reversed for 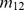. The intercept and possible covariate terms are not shown. Note the main effects (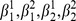) are unchanged in the reparametrization.

Advances in genomic and phenotypic technologies have vastly expanded the scope of genetic studies. Multidimensional phenotyping and high density genotyping are routinely combined to produce highly detailed views of biological systems. Translating these data into predictive models of health and disease will require new analytical methods to understand how genetic variants combine to influence multiple phenotypes. Here we present an R-based software application to use the information in multiple phenotypes to interpret statistical epistasis. The method derives models of genetic interactions as quantitative variant-to-variant and variant-to-phenotype influences. By fitting models to multiple phenotypes simultaneously, the method constrains possibilities for how individual variants interact to affect each phenotype. The result is a directed network of quantitative, variant-to-variant influences that represent specific levels of suppression or enhancement (Figure 1). The application is suitable to data sets involving engineered genetic perturbations, genetic intercross populations, and natural outbred populations containing multiple common variants. We demonstrate R/cape here using data from a mouse backcross study [1].

## Design and Implementation

R/cape is designed to detect and interpret pleiotropy and statistical epistasis. The user begins by loading their data in R/qtl format [2]. Briefly, this format encodes samples in rows and phenotype and genotype values in columns. R/cape requires a minimum of two quantitative phenotypes along with any potential covariates such as sex, lineage, or experimental treatment. The user identifies the potential covariates that are thereafter coded as genetic markers. These covariates can subsequently be included in the interaction analysis, following the same procedure as any marker. The phenotypes should exhibit limited pleiotropy, with some shared QTL but incomplete correlation, and be involved in the same high-level biological process (e.g. metabolism). Complex traits with moderate Pearson correlation (0.4≤|*r*|≤0.8) are ideal.

**Figure 2:**
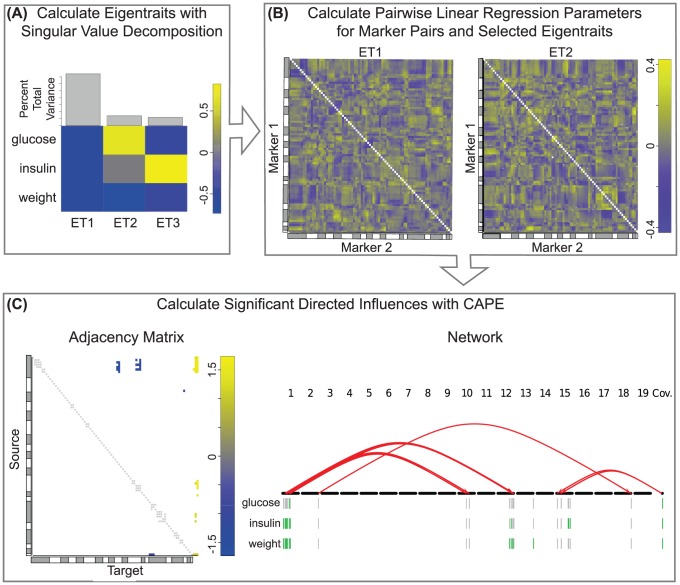
Overview of R/cape workflow and visualization tools using example data [1]. (A) Phenotypes are first decomposed into orthogonal eigentraits (ETs). Phenotype composition and global variance fraction are displayed for each ET, facilitating the selection of ETs for interaction analysis. In this study, the first two ETs were selected, which contained the correlated signal between all phenotypes and a divergence between phenotypes, respectively. (B) Pair-wise linear regression is next performed on each ET. Symmetric matrices of all marker pair interaction terms are displayed in matrix form, with gray and white bars along the axes to mark chromosome boundaries. The first two ETs for this study are shown. (C) Regression parameters are next reparametrized (Figure 1) to derive models of directed interactions between markers and from markers to phenotypes. In the adjacency matrix view (left), markers are designated as sources or targets of directed interactions, and marker-to-phenotype influences are in the rightmost columns. Only variants with significant main effect or interaction are shown, and gray dots mark pairs that were not included in the model due to linkage disequilibrium. In the network view (right), arrows are directed from source to target marker positions across all chromosomes. Red arrows indicate suppressive (negative) interactions. Main effects are represented below the variants with green indicating an effect that increases phenotype and gray indicating no significant main effect on phenotype.

To maximize linear independence of phenotypes, R/cape by default performs singular value decomposition (SVD) on two or more selected phenotypes to derive eigentraits (ETs). R/cape includes functions to visualize the percent variance accounted for by each ET as well as their individual contributions to the phenotypes, thereby aiding the selection of ETs on which subsequent analysis should be performed. Figure 2A shows the ET decomposition of the example data set (Results). By default R/cape selects the first two ETs, which typically contain the most relevant phenotypic variation, and in this example we used the default settings. For very high-dimensional phenotype data it is possible to analyze up to 12 ETs. It should be noted, however, that while increasing the number of ETs increases the information available to the algorithm, the additional information will likely contain components that may be difficult to consistently fit with single pair-wise models (Figure 1). The addition of more ETs that represent either biological signal or experimental noise will potentially reduce the significance of directed genetic interactions, as was found in a study of interacting variants that affected multiple patterns of global gene expression [3].

After ET selection, the user performs a genome-wide single-variant scan to assess the association of each individual marker. Since very strong main-effect markers can obscure interactions between other marker pairs, the user can assign covariates for the pair-scans that follow. The single-variant scan can also be used to specify marker pairs to test for interactions. The default setting is to test all possible pairs that are not in linkage disequilibrium. However, for data sets with a large number of markers, a subset of loci can be selected based on single-locus significance. Pair-scans are performed for each ET by multivariate linear regression, with an intercept, covariates, main effects, and interaction term for each pair of markers. Interaction (epistasis) coefficients from each ET can be plotted as shown in Figure 2B.

The regression coefficients are the basis for the R/cape reparametrization that determines variant-to-variant influences (Figure 1). Main effects coefficients from the pair-scans constitute the variant-to-phenotype influences [3]. For two ETs, the reparametrization is exact and the fit residuals are unchanged. For 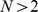 ETs, the *N* interaction coefficients are dimensionally reduced to two variant-to-variant influences (Figure 1) [3]. Significance is determined by standardized effect size, with thresholds estimated by permutation tests. Correction of p-values for multiple testing is implemented by the Holm step-down procedure [4], false discovery rate (FDR) [5], or local false discovery rate (lFDR) [6]. Null distributions are constructed by pooling all standardized effects from each permutation, with separate distributions for variant-to-variant and variant-to-phenotype distributions. This pooling procedure saves substantial computational effort and produces significance estimates indistinguishable from estimates based on distinct null distributions for every individual parameter. The final result is a directed network indicating how “source” variants influence “target” variants and how variants influence phenotypes. This directed network is represented by an asymmetric adjacency matrix with source variants in rows and target variants and phenotypes in columns. This matrix can be visualized to help identify patterns in variant-variant influences or plotted as a network (Figure 2C) with directed interactions plotted as arrows and main effects plotted as colored bars below the chromosomes. In the network, green and red denote positive and negative values, respectively.

## Results

To demonstrate the use and capabilities of R/cape, we analyzed a mouse study of obesity and type 2 diabetes [1]. This study backrossed the diabetes-prone New Zealand Obese (NZO) inbred mouse with the Non-Obese, Non-diabetic (NON) inbred strain. Body weight, serum glucose, and serum insulin were measured. All 203 male mice were genotyped at 83 autosomal microsatellite markers, with the presence of a NZO allele coded as the perturbation at each locus. Maternal obesity, here coded as “mom,” was used as a covariate and assessed for interactions with genetic variants. The data provided a system in which R/cape analysis could exploit both the identical and distinct factors contributing to obesity, glucose levels, and insulin regulation in deriving a polygenic model [7], [8]. After normalization, all phenotypes exhibited Pearson correlation coefficients around 0.6.

Phenotypes were decomposed into uncorrelated eigentraits (ETs). Phenotype contributions to each ET are shown in Figure 2A. The first ET represented the common, correlated signal for all traits, while the second encodes the divergence between plasma glucose and body weight. These two ET were selected for R/cape analysis, and their pair-wise regression results are shown in Figure 2B. These symmetric, marker pair regression results were reparametrized (Figure 1) to derive directed influences from source markers to target markers (Figure 2C).

R/cape analysis was then performed on the selected ETs. Two markers were excluded due to full redundancy with adjacent markers and individual pairs with substantial linkage disequilibrium (LD) were not tested, defined as fewer than six instances of the four possible pair-wise genotypes at the two loci. These pairs are marked by gray dots in the pairscan results (Figure 2C). We identified both influences of markers on phenotypes as well as directed interactions between markers using a false discovery rate of 0.01. NZO variants on multiple chromosomes show positive effects on all traits (Figure 2C), denoting increased body weight and diabetes risk. The effects between alleles, on the other hand, are uniformly negative, which may indicate canalization of these traits in the inbred founder lines. Maternal obesity was found to suppress the effects of markers on Chromosome 15, and a marker on Chromosome 2 suppressed the effects of a marker on Chromosome 18. In the latter interaction, neither marker had an individual main effect, although the Chromosome 18 marker has a marginally significant effect (q = 0.018) on body weight that is completely suppressed by the Chromosome 2 marker. NZO variants in a region on Chromosome 1 increased all three phenotypes individually and were the hub of a small epistatic network suppressing the effects of markers on Chromosomes 10 and 12.

**Figure 3:**
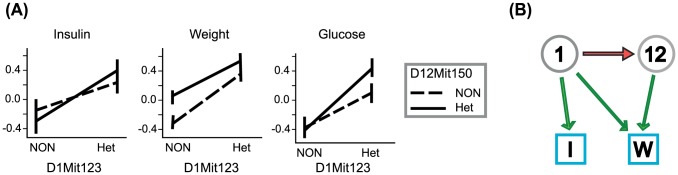
R/cape-derived interaction between Chromosome 1 and Chromosome 12. (A) R/cape effect plots showing normalized phenotype values for each combination of the Chromosome 1 (D1Mit123) and Chromosome 12 (D12Mit150) genotypes. NON denotes a homozygous locus and Het denotes an NON/NZO heterozygote. Positive slopes correspond to Chromosome 1 effects, while differences between solid and dashed lines represent the Chromosome 12 effects. Positive and significant weight effects were detected for both loci, whereas effects on insulin were only significant for the Chromosome 1 marker. Neither locus had a significant effect on serum glucose. Significant epistasis was detected for weight, and appears as the convergence of solid and dashed lines for heterozygosity at the Chromosome 1 locus. Note that plotted data are not conditioned on maternal obesity, which was a covariate in the analysis. (B) Interaction network for variants on Chromosomes 1 and 12, extracted from Figure 2C, showing the significant suppressive interaction (red arrow) from the Chromosome 1 variant to the Chromosome 12 variant (gray circular nodes). Significant main effects (green arrows) link the source variants with the target phenotypes weight and insulin (square nodes labeled “I” and “W” respectively).

The interaction between Chromosomes 1 and 12 is illustrative of the R/cape strategy (Figure 3). Although both loci have a positive effects on body weight, their joint effect is less than additive (Figure 3A, center plot). This suggests that one or both of the loci are suppressing the effect of the other, but is ambiguous about the direction of suppression. In contrast, only the Chromosome 1 locus has an effect on insulin and this effect is independent of the Chromosome 12 locus (Figure 3A, left plot). This second phenotype provides R/cape the information necessary to infer the directionality of the interaction from Chromosome 1 to 12, since a reversed interaction would imply epistasis for insulin along with body weight. Specifically, if the Chromosome 12 locus suppressed the Chromosome 1 locus effect, we would expect to see the solid line in the insulin plot to have a reduced slope, since the presence of heterozygosity at Chromosome 12 would reduce the effect of a heterozygous allele at Chromosome 1. This was not observed. R/cape thus provides a more stringent hypothesis for gene candidates in the two loci through the constraint of directional genetic effects.

The interacting loci we detected were distinct from those reported in the original analysis [1], in which phenotypes were separately tested for evidence of interaction. The discrepancy is a result of R/cape integrating signals across multiple phenotypes. R/cape interactions may be of low significance for any single phenotype, and therefore not detected in the previous study. Furthermore, the integration strategy requires differential information across multiple phenotypes, so the instances of redundant epistasis reported in [1] were not detected with R/cape. R/cape also uses permutation testing and conservative correction for multiple testing to limit the discovery of false positive interactions. Permutation testing in particular guards against false positives arising from linkage between markers.

By simultaneously anlayzing multiple phenotypes, R/cape can reveal complex interactions between genotype and phenotype not seen when examining phenotypes in isolation. While the original study was based on detecting loci involved in the composite phenotype “diabesity,” R/cape parses how genetic loci both jointly and distinctly affect diabetes and obesity. This is of interest to geneticists because, although these traits are correlated, not all obese individuals are diabetic and not all diabetic individuals are obese [7], [8]. The effects of one locus can be direct or indirectly mediated by the genotype at a second locus, as mapped by our algorithm. Parsing how genetic factors directly and indirectly affect these phenotypes provides a clearer model of how certain loci are more relevant to the diabetic state. As genetic studies gain power through larger sample sizes, improved population design, and increased phenotype precision, the R/cape algorithm is designed to parse multiple genetic loci into interacting subnetworks of variants that differentially affect a number of related traits [9].

Together these findings illustrate how R/cape is designed to find interactions that simultaneously model all phenotypes under the assumption that interactions between variants across multiple contexts represent a single underlying interaction network. We note that in a large-scale, highly-powered study based on genetic interaction screens R/cape analysis yielded significant interactions for 55% of the interactions identified as significant using standard epistasis analysis [9]. Thus we recommend users assess single-phenotype epistasis using functions in R/cape or in parallel analyses using tools such as R/qtl and R/qtlbim [10] as the results are expected to be somewhat complementary.

## Availability and Future Directions

The R/cape package is freely available under the GPL-3 license at the Comprehensive R Archive Network (CRAN), http://cran.r-project.org/web/packages/cape. The R platform and all R dependencies are similarly available from CRAN.

The time required to perform an R/cape analysis depends on multiple parameters including the number of markers, the number of phenotypes, and the amount of linkage between markers since linked marker pairs are not tested. Overall processor time is proportional to 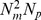, where 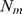 is the number of markers tested and 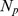 is the number of permutations performed. The analysis reported here used 83 markers and one covariate, generating 3463 pairs (after exclusion of markers in LD). Each pair was tested with 100 permutations. On an iMac with a 2.7 GHz Intel processor and 8 GB of memory the full analysis took 5.5 hours. Since permutation results are pooled to generate null distributions, preliminary analyses can be performed with fewer permutations in order to save substantial processing time. Optional limits are implemented for the number of pairs and permutations passed to the main analysis functions.

Future additions to R/cape will include advanced graphics capabilities and additional features to manage populations. We intend to augment the current graphic with scalable graphs. Application of the software to genome-wide association studies is currently possible but requires quantitative data and care in managing population structure. Corrections for sample relatedness are under development. Finally, we will expand allele encoding to address more than two alleles at each locus, based on precision genotyping or inferred haplotype probabilities. This will enable application of R/cape to data from the Collaborative Cross [11], Diversity Outbred [12], Heterogeneous Stock [13], and similar resources.

In conclusion, R/cape is an efficient tool to represent genetic complexity in terms of models of variant action. The program will aid in the interpretation of epistasis without prior knowledge by providing a fast overview and hypothesis generation tool.

## Supporting Information

Software S1 **R/cape software package.** Source code, documentation, test data, and an instructive vignette for R/cape.
(GZ) Click here for additional data file.
