# Supplementary material for: CAPE: An R Package for Combined Analysis of Pleiotropy and Epistasis
Source: PLoS Comput Biol. 2013 Oct 24;9(10):e1003270. doi: 10.1371/journal.pcbi.1003270 (PMC3808451; doi:10.1371/journal.pcbi.1003270)
Supplement: Software S1 — R/cape software package. Source code, documentation, test data, and an instructive vignette for R/cape. (GZ) [file pcbi.1003270.s001.gz › cape/inst/doc/cape.pdf]

cape: A package for the combined analysis of epistasis and pleiotropy

Anna L. Tyler[1], Wei Lu[1], 2, Justin J. Hendrick[1], Vivek M. Philip[1], and Gregory W. Carter[1]

July 25, 2013

[1]The Jackson Laboratory, Bar Harbor, ME, 04609 [2]Department of Electrical and Computer Engineering, Duke University, Durham, NC 27708

Contents

Abstract **1**

Loading Data **2**

Manipulating the Data Object **4**

Looking at the Data **4**

Decomposing the Phenotypes **8**

Single-Variant Scan **10**

Covariate Selection **12**

Pairwise Scan **14**

Combined Analysis for Detection of Interactions **18**

Interpretation of Results **23**

Tables of Functions **26**

Abstract

Here we present an R package for the Combined Analysis of Epistasis and Pleiotropy, or `cape`. This package implements a method, originally described in Carter et al. (2012), that infers directed interaction networks between genetic variants for predicting the influence of genetic perturbations on phenotypes. This method takes advantage of complementary

information in partially pleiotropic genetic variants to resolve directional influences between variants that interact epistatically. `cape` can be applied to a variety of genetic variants, such as single nucleotide polymorphisms (SNPs), copy number variations (CNVs) or structural variations (SVs). Here we demonstrate the functionality of `cape` by inferring a predictive network between quantitative trait loci (QTL) in a cross between the non-obese, non-diabetic (NON) mouse and the New Zealand obese (NZO) mouse (Reifsnyder, 2000).

Loading Data

For the purposes of demonstration, we will reanalyze a data set described in Reifsnyder (2000). This data set was established to find quantitative trait loci (QTL) for obesity and other risk factors of type II diabetes in a reciprocal back-cross of non-obese non-diabetic NON/Lt mice and diabetes-prone, New Zealand obese (NZO/HILt) mice. The study found multiple main-effect QTL influencing phenotypes associated with diabetes and obesity as well as multiple epistatic interactions. In addition, maternal environment (i.e. whether the mother was obese) was found to interact with several markers and epistatic pairs to influence the risk of obesity and diabetes of the offspring. The complex nature of diabetes and obesity, along with their complex and polygenic inheritance patterns, make this data set ideal for an analysis of epistasis and pleiotropy.

Included in this dataset are 204 male mice genotyped at 85 markers across the genome. The phenotypes included are the body weight (g), insulin levels (ng/mL), and plasma glucose levels (mg/dL), all measured at age 24 weeks. In addition, there is a variable called "mom" indicating whether the mother of each mouse was normal weight (0) or obese (1). After installing `cape`, load the package and the dataset type the following in the R command line.

```
> library(cape)
> data(obesity.cross)
```

To load your own data, use the function `read.population()`. This function is similar to the function `read.cross()` used in the R package `qtl` (Broman et al., 2003), and accepts the basic `R/qtl` CSV format. For more information about this function type `?read.population`. In this function the data file can be specified as an argument.

```
> obesity.cross <- read.population("Obesity.Cross.csv")
```

Alternatively, the filename can be left blank to choose a file through the file system. The following code will bring up a window for choosing the desired file:

```
> obesity.cross <- read.population()
```

If the phenotypes are not specified in `read.population()` the default behavior is to read in all phenotypes. The functions `delete.pheno()` and `select.pheno()` can be used after reading in the data to narrow down the phenotypes for analysis. For `cape` analysis there must be between two and 12 phenotypes. At least two phenotypes must be used because the

fundamental concept of cape uses partial pleiotropy to refine models of epistasis. However, too many phenotypes may reduce power to detect interactions.

Upon being read in, the data are formatted and stored in an object. This object is referred to as data.obj in the argument lists of most functions in cape. The main functions in this analysis return data.obj with any results appended to it. The structure of this object can be viewed at any time by using the core R function str(). For example, the data that we have just loaded is named obesity.cross, and its structure can be seen here:

```
> str(obesity.cross)
```

```
List of 5
 $ pheno          : num [1:204, 1:4] 58.6 49.9 56 53.7 48.7 41.1 45.4 44.1 40.4 40.1 ...
  ..- attr(*, "dimnames")=List of 2
  .. ..$ : NULL
  .. ..$ : chr [1:4] "body_weight" "glucose" "insulin" "mom"
 $ geno           : num [1:204, 1:85] 0.5 0.5 0.5 0 0.5 0 0.5 NA 0 0 ...
  ..- attr(*, "dimnames")=List of 2
  .. ..$ : NULL
  .. ..$ : chr [1:85] "1" "2" "3" "4" ...
 $ chromosome     : chr [1:85] "1" "1" "1" "1" ...
 $ marker.names   : chr [1:85] "D1Mit296" "D1Mit211" "D1Mit411" "D1Mit123" ...
 $ marker.location: num [1:85] 2.08 10.59 12.62 17.67 22.88 ...
```

Individual elements of this list can be accessed by name by using the following syntax:

```
data.obj$name
```

When the cross is first loaded into cape it contains the following elements:

- **$pheno** - The phenotype matrix in which individuals are in rows and phenotype values are in columns.
- **$geno** - The genotype matrix in which individuals are in rows and genetic loci are in columns. Each cell of the genotype matrix contains the probability that individual i has the reference allele at that position. By default, the reference allele is the allele that is alphabetically first if the original data file used letter designations for genotypes, or "0" if the original data file used numeric genotype designations. In the example backcross, NON/Lt is the reference allele and the heterozygote state is coded as the perturbation. Thus cape will model the effects of NZO/HILt variants.
- **$chromosome** - The chromosome on which each genetic locus is situated
- **$marker.names** - The alphanumeric names of markers.
- **$marker.location** - The position of each locus on each chromosome

Most functions performed on data.obj will add elements to this list containing the results of the analyses.

Manipulating the Data Object

There are a number of functions available for simple manipulation of the data object. For example, after the data have been read in, phenotypes may be removed from the phenotype matrix using `delete.pheno()`. To select specific phenotypes, use the function `select.pheno()`.

```
> obesity.cross <- select.pheno(obesity.cross,
+ phenotypes = c("body_weight", "glucose", "insulin", "mom"))
```

In `cape` covariates are imported as phenotypes and must be reassigned as genetic markers. To code a variable, such as sex, experimental treatment, or another environmental variable, as a covariate, use the function `create.covar()` to transfer a variable from the phenotype matrix to the genotype matrix. Here we convert the factor maternal environment ("mom") to a covariate.

```
> obesity.cross <- create.covar(obesity.cross, "mom")
```

The data object can also be subset by chromosome (`select.by.chr()`) or by individual (`select.by.ind()`) before the analysis if desired. Individuals can be selected based either on phenotype or genotype value. For example, to use only individuals with a plasma insulin level less than than 25 ng/mL:

```
> obesity.cross <- select.by.ind(obesity.cross, "pheno", "insulin < 25")
```

Finally, it is also possible to remove all individuals from the cross with missing phenotype data (`remove.ind.with.missing.pheno()`).

Examining the Data

Before proceeding with an analysis it is recommended that the data be examined by eye. The R package `qtl` has sophisticated plotting tools for examining genetic cross data, which we do not try to duplicate here. It is straightforward enough, however, to examine phenotype distributions for normality, batch effects and other quality control issues using standard R functions. Using the function `hist()`, we can look as the distribution of each phenotype:

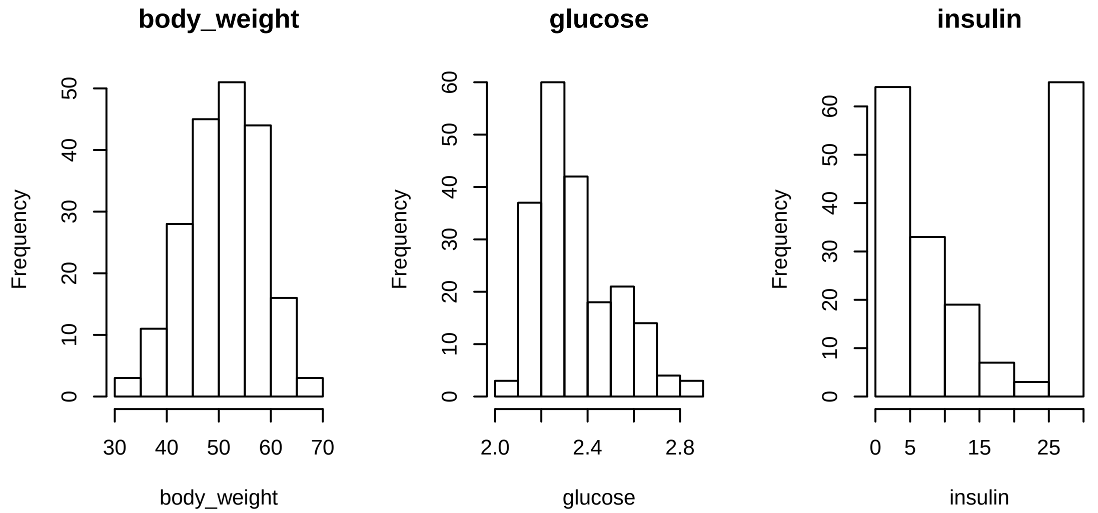

While body weight looks relatively normally distributed, glucose and insulin have obviously non-normal distributions.

Examination of the Q-Q plots of pairs of phenotypes, using the core R function `qqplot()`, can reveal phenotyping errors and other pathologies. Here we see a threshold and ceiling effect in the relationship between the distributions of insulin and the other two phenotypes.

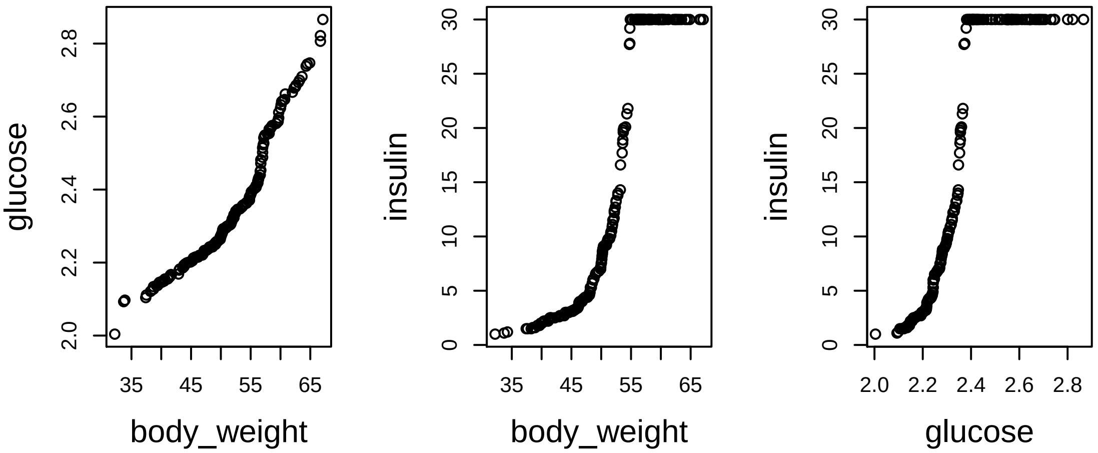

In general we recommend mean centering and normalizing all phenotypes before proceeding with the analysis. Phenotype normalization can be achieved through log transformation, quantile normalization, or another method before the analysis. The function `norm.pheno()` uses quantile normalization to fit the phenotypes to a normal distribution. Briefly, this process sorts the values of the phenotype and replaces each with a corresponding value drawn from a normal distribution with the same standard deviation and mean as the original distribution. Mean centering subtracts the mean phenotype value from each phenotype value yielding a distribution centered around 0.

```
> obesity.cross <- norm.pheno(obesity.cross, mean.center = TRUE)
```

Plotting the histograms of the normalized data confirms the normalization. Only insulin still has a ceiling effect, which cannot be removed by normalization because rank cannot be determined among equal values.

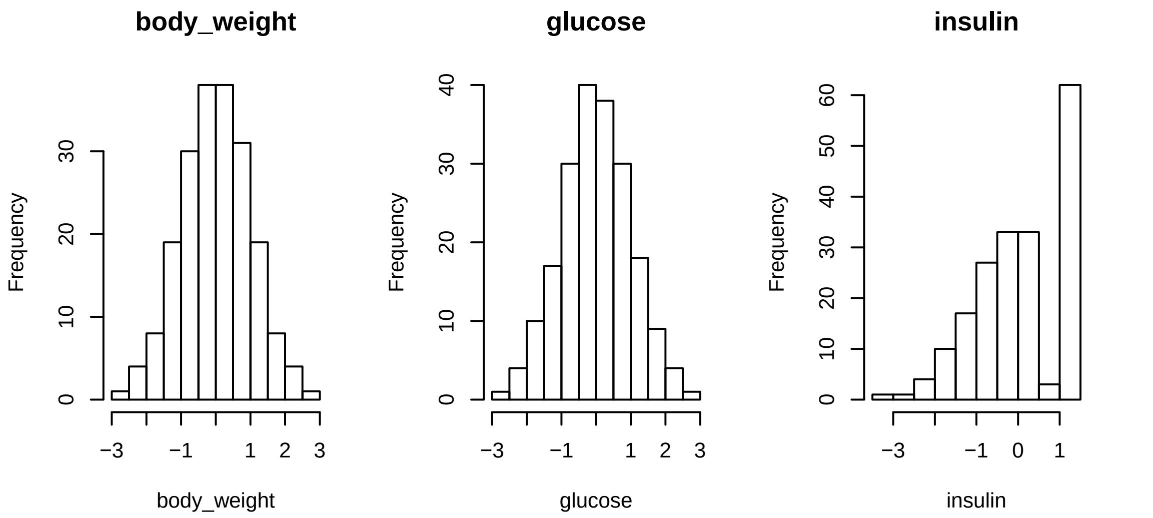

We can also see that the Q-Q plots from before show the example phenotypes have been converted to the same distribution. The ceiling effect is still visible in the insulin measurement, but this cannot be removed through normalization, and overall, the distribution is more similar to those of the other phenotypes than before normalization. Knowing that this ceiling effect is present will be important in interpreting the results of the analysis.

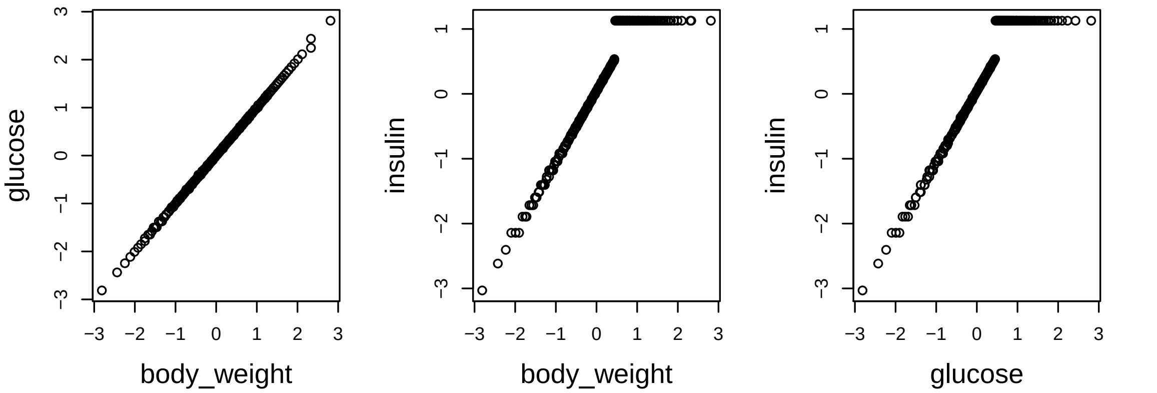

At this point, if we decide to exclude insulin from the analysis because its distribution cannot be normalized, we can simply remove it from the data object using `delete.pheno()`.

```
> obesity.cross <- delete.pheno(obesity.cross, phenotypes = "insulin")
```

For the example here, however, we will include it in the analysis.

A Note on Phenotype Selection

Phenotype selection is an important component of the `cape` analysis and should be given considerable thought. This method relies on the selection of two or more phenotypes that have common genetic factors but are not identical across all individuals. Such phenotypes may describe multiple aspects of a single complex trait, such as obesity or diabetes, and may encompass a combination of molecular phenotypes, such as plasma glucose levels, and phenotypes, such as body weight, that are measured at the organismal level. The central assumption of this method is that different genetic interactions found for a single gene pair in the context of different phenotypes represent multiple manifestations of a single underlying gene network. By measuring the interactions between genetic variants in different contexts we can gain a clearer picture of the network underlying statistical epistasis (Carter et al., 2012).

The phenotypes in the Reifsnyder (2000) data set are ideal for the `cape` analysis. They measure different aspects of the diabetes and obesity, two complex traits that are known to be related biologically and are highly correlated. The phenotypes themselves range from molecular phenotypes to organismal phenotypes. By examining the correlations between phenotypes, we can see that the phenotypes measured in this experiment are correlated, but not identical across all individuals. Ideally, phenotypes used in cape should have a Pearson correlation coefficient r between 0.4 and 0.8.

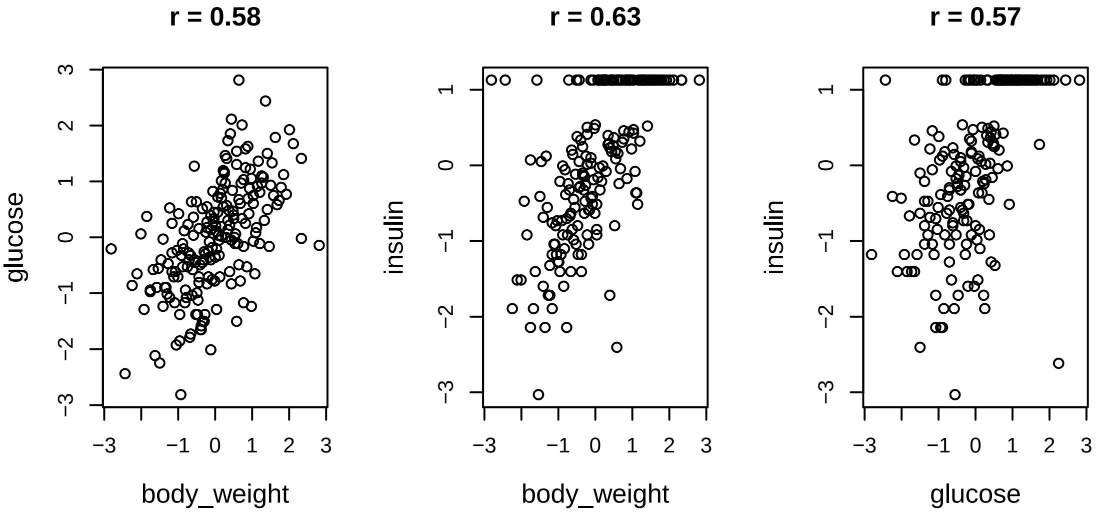

Note that all mice in the example backcross are male. For multisex populations, sex is usually a covariate and correlation should be assessed for each sex separately.

Decomposing the Phenotypes

Although `cape` can find genetic variants associated with raw phenotypes, the analysis was designed to work on composite traits called "eigentraits." Eigentraits are calculated by factoring the matrix of phenotypes by singular value decomposition (SVD):

$$Y = U \cdot V \cdot W^T$$

Where Y is a matrix containing one column for each mean-centered, normalized phenotype and one row for each individual. If Y contains more individuals than phenotypes, the U matrix has the same dimensions as Y with each column containing one eigentrait. V contains the singular values, and W^T contains the right singular vectors in rows. See Carter et al. (2012) for more details.

The SVD de-correlates the phenotypes concentrating phenotypic features into individual eigentraits. One benefit of this process is that variants that are weakly correlated to several phenotypes due to common underlying processes may be strongly correlated to one of the eigentraits. This eigentrait captures the information of the underlying process, making strong main effects distributed between phenotypes easier to detect and identify as potential interaction loci and/or covariates. Thus, analysis of eigentraits is recommended over the analysis of raw traits.

To decompose the phenotypes to eigentraits use the function `get.eigentraits()` If the phenotypes have not already been mean centered and normalized, this step can be performed here. In this example, we do not carry out these steps, as they have already been performed.

```
> obesity.cross <- get.eigentraits(obesity.cross, scale.pheno = FALSE,
+ normalize.pheno = FALSE)
```

This function performs the SVD and returns the matrix of eigentraits in a new element of the list called `ET`. The result of the decomposition can be viewed with the function `plotSVD()`.

```
> plotSVD(obesity.cross, orientation = "vertical")
```

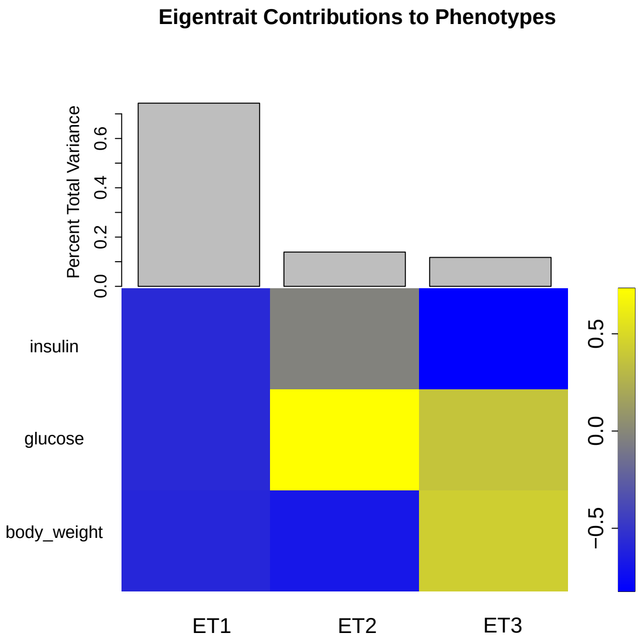

In the example illustrated here, the first eigentrait captures more than 70% of the variance in the three phenotypes. This eigentrait describes the processes by which body weight, glucose levels, and insulin levels all vary together. The correlations between obesity and risk factors for obesity, such as elevated insulin and fasting glucose levels are well known (Permutt et al., 2005; Das and Elbein, 2006; Haffner, 2003). The second eigentrait captures nearly 20% of the variance in the phenotypes. It captures the processes through which glucose and body weight vary in opposite directions. This eigentrait may be important in distinguishing the genetic discordance between obesity and diabetes. While obesity is a strong risk factor for diabetes, not all those who are obese have diabetes, and not all those with diabetes are obese (Permutt et al., 2005; Burcelin et al., 2002).

The third eigentrait is less interpretable biologically, as it describes the divergence of blood glucose and insulin levels. It may represent a genetic link between glucose and body weight that is non-insulin dependent. Because we are primarily interested in the connection between diabetes and insulin, we will use only the first two eigentraits for the analysis. In many cases in which more than two phenotypes are being analyzed, the first two or three eigentraits will capture the majority of the variance in the data and capture obvious features. Other eigentraits may capture noise or systematic bias in the data. Often the amount of total variance captured by such eigentraits is small, and they can be removed from the analysis.

Ultimately, there is no universal recipe for selecting which eigentraits should be included in the analysis, and the decision will be based on how the eigentraits contribute to the original phenotypes and how much variance in the data they capture.

To select eigentraits for the analysis, use the function `select.eigentraits()`. Here we select the first two eigentraits.

```
> obesity.cross <- select.eigentraits(obesity.cross, traits.which = c(1,2))
```

Single-Variant Scan

Once the eigentraits for the analysis have been selected, the single-locus scan is run to investigate how individual markers are associated with each eigentrait. Note that this scan performs a linear regression at each marker. A more sophisticated single-locus analysis can be performed by `R/qtl` (Broman et al., 2003). This single-marker scan in `cape` performs the following regression for each locus on each eigentrait:

$$U_i^j = \beta_0^j + x_i \beta^j + \epsilon_i^j$$

The index i runs from 1 to the number of individuals, and j runs from 1 to the number of eigentraits or phenotypes. x_i is the probability of the presence of the reference allele for individual i at locus j. The purpose of this scan is two-fold:

- In large data sets the number of possible variant pairs may be too large to test exhaustively. The single-variant scan can be used as a filtering step to choose variants that will be included in the pair scan.
- Large main effects can obscure interactions. In sufficiently powered studies, conditioning on the large QTL can aid in the discovery of interactions, variants with large main-effects can be used as covariates in the pair scan.

The function `singlescan()` runs the single-marker scan and has several arguments associated with selecting markers to include in the pairwise scan as well as for selecting markers to use as covariates in the pairwise scan.

```
> obesity.cross <- singlescan(obesity.cross, n.perm = 100, covar = "mom",
+ scan.what = "eigentraits", auto.covar.selection = FALSE,
+ alpha.for.covar = 0.01, alpha.for.pairs = 0.1, verbose = FALSE)
```

Markers can be selected for inclusion in the pairwise scan based on their having significant main effects as determined through permutation testing. The significance threshold is set with the argument `alpha.for.pairs`. After the single-locus scan, if filtering markers by significance is desired, the function `select.markers.for.pairscan` selects only those markers exceeding this significance cutoff for inclusion in the pairscan. If no filtering is desired, this threshold is ignored.

The function `singlescan()` can also automatically select markers to include as covariates in the pairwise scan. Like the marker inclusion selection, marker covariate selection is also based on marker effect size, and the alpha for this threshold is set by the argument `alpha.for.covar`. Automatic covariate selection is recommended in populations in which genetic markers are independent of one another, but not in populations in which markers are linked (see Covariate Selection). To use automatic covariate selection, set the argument `auto.covar.selection` to `TRUE`.

Covariates to be used in the single-marker scan should be specified through the argument `covar`. These covariates should have already been moved to the marker matrix by `create.covar()`. An additional argument `scan.what` determines whether "eigentraits" or "raw.traits" will be used as the dependent variable in the regression.

The single-marker scan currently does not support markers on sex chromosomes. Because the X chromosome is hemizygous in males, sex differences in phenotype can lead to false associations, and markers on this chromosome require special consideration (Broman et al., 2006). Before the single-marker scan is performed, any markers on the X and Y chromosomes are removed from the data object. The results of the single variant scan can be visualized with the function `plotSinglescan()`.

```
> plotSinglescan(obesity.cross, mark.chr = TRUE, mark.covar = FALSE)
```

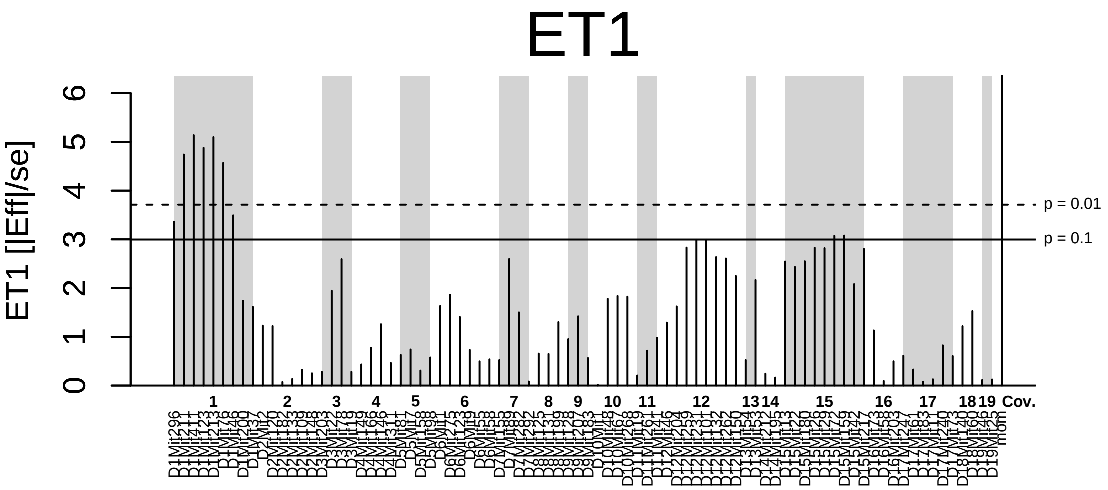

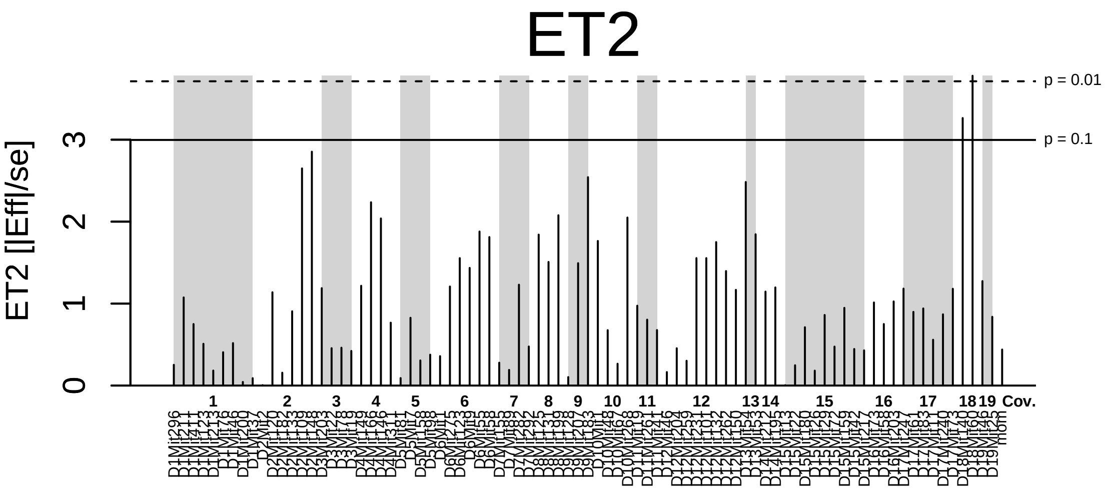

In this figure the t-statistic (β/σ) of each marker is plotted as a vertical line. Results for both eigentraits are shown here as ET1 and ET2, and chromosome numbers are written along the x axis. If markers were being filtered for inclusion in the pair scan, the markers above the covariate line at p = 0.01 would be selected for inclusion in the pair scan. The alpha value can be adjusted to include more or fewer markers in the pair scan as desired. The value of this threshold is stored in `obesity.cross$pairscan.thresh` and can also be assigned manually using `set.pairscan.thresh()`. This threshold is useful for large data sets for which it is impractical to test all marker pairs. Here we use all marker in the pairwise scan. In this example we will not filter the markers.

The higher dashed line represents the alpha value defined by the argument `alpha.for.covar`, which here was set to the default value of 0.01. This threshold can be used for automatic covariate selection. If the argument `automatic.covar.selection` is set to `TRUE` all markers exceeding the `alpha.for.covar` threshold will automatically be designated as covariates. This automated selection is generally only recommended for populations in which variants segregate independently. For more details on covariate selection, see Covariate Selection.

Covariate Selection

As mentioned previously, conditioning on large main effects may aid in the discovery of interactions. Therefore `cape` allows specification of genetic markers as covariates, which can be done either manually or automatically. Automatic covariate selection is particularly useful in populations in which variants segregate independently. For example, *Drosophila* have virtually no linkage between markers (Mackay et al., 2012); therefore most markers can be assumed to assort independently. Similarly, mutations to genes on different chromosomes in a yeast population segregate independently. In these and other similar cases, automatic covariate selection is appropriate because markers achieving the significance threshold can be assumed to be independent of each other and will not reduce the significance of other markers due to linkage if used as covariates.

In a mouse F2 intercross or backcross, on the other hand, markers on a single chromosome tend to be linked, and groups of markers may be significantly associated with a phenotype due to linkage with a causative locus. In this case, markers from a single linkage block carry partially redundant information and, if treated as independent covariates, will obfuscate interactions between makers in the linkage block and other truly independent markers. Thus, if markers in the study population are linked, it is recommended that any marker covariates be set manually and that only one covariate should be selected from each linkage block.

To use automatic covariate selection, set `auto.covar.selection` to `TRUE` when running `singlescan`. All markers above the `alpha.for.covar` threshold will be automatically included as covariates in the pair scan. If this option is chosen, is is still possible to adjust the covariate threshold after the single-marker scan to include more or fewer covariates as desired. In general we recommend that only the markers with the very strongest effects are treated as covariates to avoid overfitting the model. The function `get.covar()` sets the covariate threshold in the data object and recalculates which markers are to be used as

covariates in the pair scan.

```
> obesity.cross <- get.covar(obesity.cross, covar.thresh = 4.5)
```

If selecting covariates manually, set `auto.covar.selection` to `FALSE` when running `singlescan`. The plots from the single-marker scan can then be examined to determine which markers should be used as covariates. The function `set.covar()` can then be used to set covariates individually.

Here, because of the large effects of maternal environment on ET1, we make this marker a covariate. The function `set.covar()` optionally includes a plot to verify the covariate selection. Covariates are marked in red.

```
> obesity.cross <- set.covar(obesity.cross, pheno = "ET1",
+ markers = c("mom"), is.covar = TRUE, plot.covar = TRUE)
```

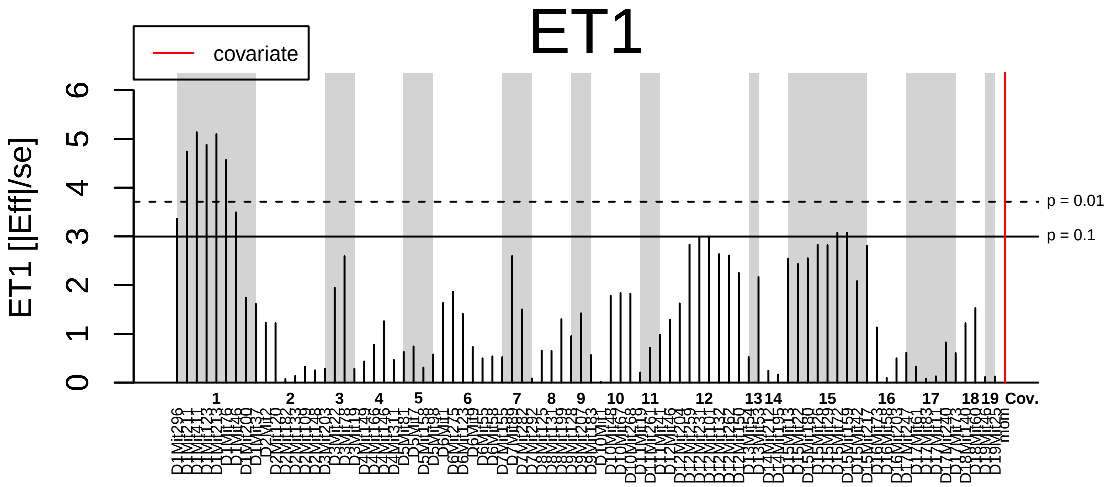

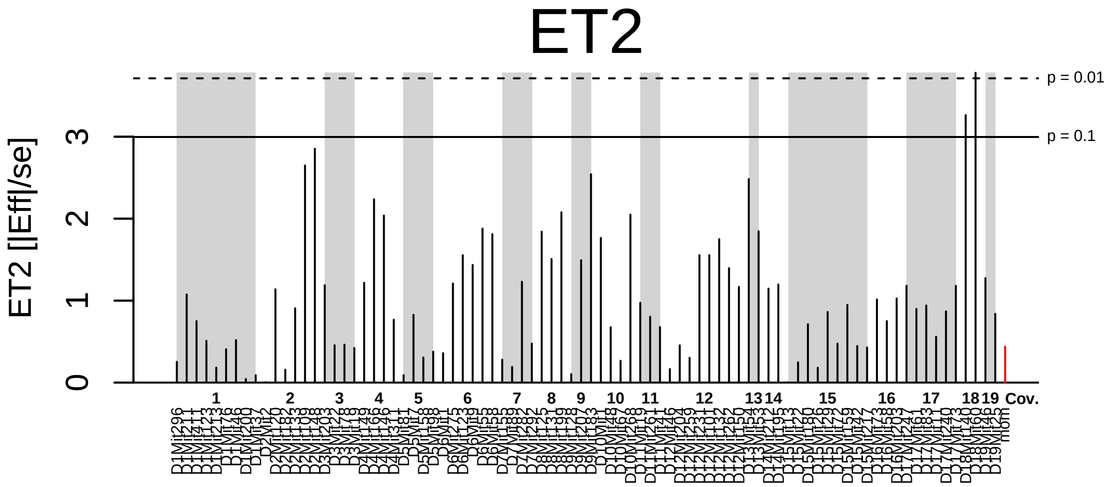

Pairwise Scan

The purpose of the pairwise scan is to find interactions, or epistasis, between variants. The epistatic models are then combined across phenotypes or eigentraits to infer a parsimonious network that takes data from all eigentraits into account.

To find epistatic interactions `pairscan()` tests the following model for each variant 1 and 2:

$$U_i^j = \beta_0^j + \underbrace{\sum_{c=1}^{2} x_{c,i}\beta_c^j}_{\text{covariates}} + \underbrace{x_{1,i}\beta_1^j + x_{2,i}\beta_2^j}_{\text{main effects}} + \underbrace{x_{1,i}x_{2,i}\beta_{12}^j}_{\text{interaction}} + \epsilon_i^j$$

The terms in this equation are the same as those in the equation for the single-variant scan except for the addition of the term for the interaction between the two variants being tested. This additional term brings further complications to the model, and restricts which markers can be tested. Because many markers, including covariates, may be included in the model, we need to be careful about including only markers that are linearly independent of each other. Linear dependence between markers occurs when two markers are fully linked and therefore perfectly correlated. The two markers provide the same genetic information, and one can be discarded without loss of information. Before running the pair scan, it is important that we reduce the genetic matrix to only markers that are linearly independent of one another. This step is performed by the function `select.markers.for.pairscan()`. It calculates the correlation between all pairs of markers. If any are found to be perfectly correlated, the first marker is removed.

`select.markers.for.pairscan()` also filters markers for inclusion in the pair scan by the significance threshold, if this has been specified by `use.pairs.threshold`. This is useful in large crosses in which it may be impossible to test all possible pairs of markers in a reasonable amount of time. The significance threshold is determined by the argument `alpha.for.pairs` in the function `singlescan()`.

```
> obesity.cross <- select.markers.for.pairscan(obesity.cross,
+ use.pairs.threshold = FALSE)
```

The number of markers removed is printed to the screen, and the names of these markers are written to the file `markers.removed.txt` in the current working directory. This filtering step adds two elements to the data object. One is a filtered genotype matrix called `geno.for.pairscan`. The other, `covar.for.pairscan`, indicates which markers are to be used as covariates in the pair scan.

The results of the filtering can be visualized with `ploSinglscan()`. This visualization can be helpful in seeing the locations of discarded markers, especially if many have been removed. To indicate which markers have been selected for the pair scan, set `show.selected.markers` to `TRUE`. To indicate which markers have been rejected from the pair scan, set `show.rejected.markers` to `TRUE`.

```
> plotSinglescan(obesity.cross, mark.chr = TRUE, show.rejected.markers = TRUE,
+ standardized = TRUE)
```

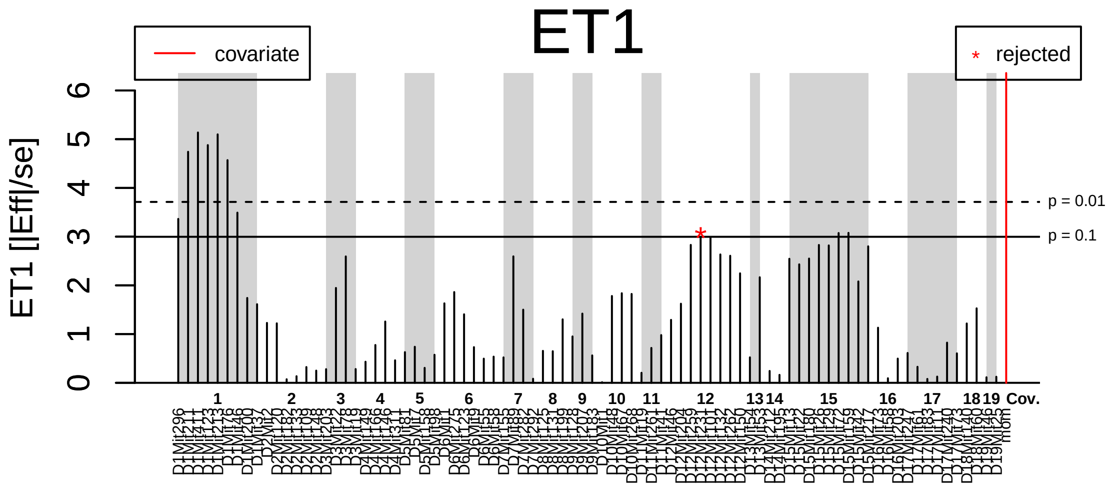

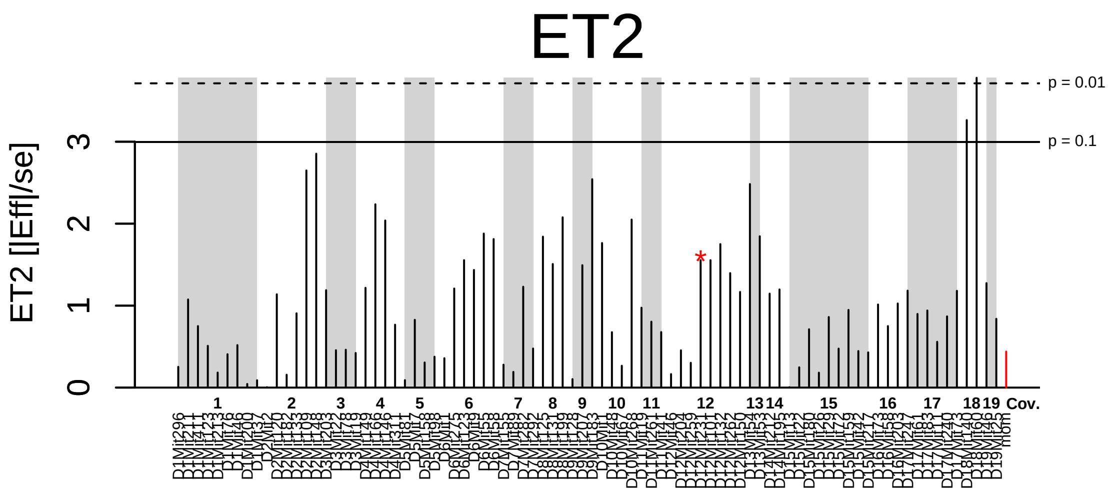

After ensuring that all markers are linearly independent and thresholded satisfactorily, the pair scan can be run using `pairscan()`.

```
obesity.cross <- pairscan(obesity.cross, scan.what = "eigentraits",
n.perm = 100, min.per.genotype = 6, verbose = FALSE)
```

The arguments here are familiar from `singlescan()`, with the exception of one additional argument. `min.per.genotype` is a threshold that prevents empty cells in genotype matrices. In an intercross, there are three possible genotypes at each marker, giving a total of nine possible genotypes for the pair. For example, for two markers each with the genotypes AA, AB, and BB, the pairwise genotypes are the following:

	AA	AB	BB
AA			
AB			
BB			

In a backcross each marker only has two possible genotypes, AA and AB, yielding four possible pairwise genotypes. In both cases, insufficient representative individuals of single genotypes indicates that the two markers in question are linked. This linkage leads to false associations in both the effects and permutations. False associations in permutations results in a heavy-tailed null distribution, which artificially inflates the threshold of significance and reduces power to find true interactions. Because of this effect, linked marker pairs are not tested. The threshold used to determine insufficient recombination between markers is given by `min.per.genotype`. The default behavior is to reject any marker pair for which there are fewer than six individuals in any of the genotype cells. This value can be adjusted, but caution should be used in interpreting the results if `min.per.genotype` is very low or 0.

The `n.perm` argument in `pairscan()` sets the number of permutations performed per marker pair. To calculate empirical p values, the permutations are combined across all markers to result in one large null distribution. Thus, large null distributions can be achieved with relatively few permutations per pair. This is useful, as even with relatively sparse genotyping the number of tests performed can be large and take many hours to perform.

The results of the pair scan can be plotted with `plotPairscan()`. This plot shows the resulting β coefficient for each pair of markers. The t statistics of each interaction (β/σ) can be plotted by setting `standardized = TRUE`. Gray and white bars show the boundaries of the chromosomes.

```
plotPairscan(obesity.cross, phenotype = c("ET1", "ET2"), standardized = FALSE,
pdf.label = "Pairscan.Regression.pdf")
```

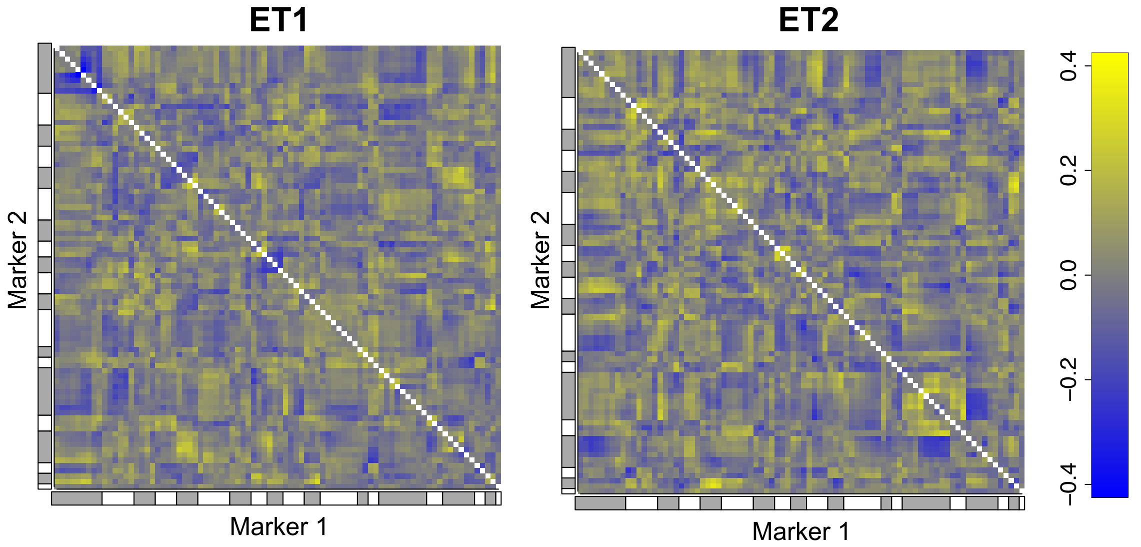

Combined Analysis for Detection of Interactions

From the pair scan, each pair of markers 1 and 2 receives a set of β coefficients describing the main effect of each marker on each eigentrait j (β_1^j and β_2^j) as well as the interaction effect of both markers on each eigentrait (β_{12}^j) (See figure below). The central idea of `cape` is that these coefficients can be combined across eigentraits and reparameterized to calculate how each pair of markers influences each other directly and independently of eigentrait.

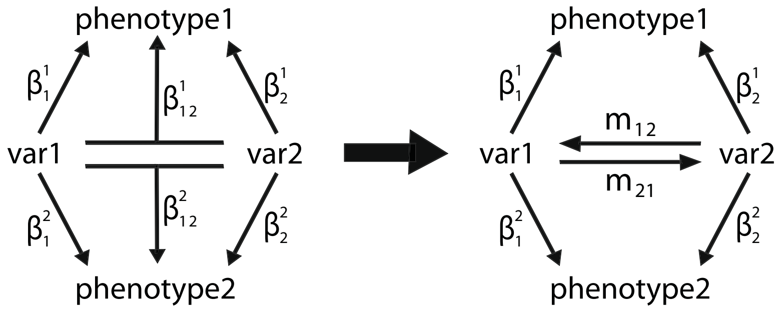

The first step in this reparameterization is to define two new parameters (δ_1 and δ_2) in terms of the interaction coefficients. δ_1 can be thought of as the additional genetic activity of marker 1 when marker 2 is present. Together the δ terms capture the interaction term, and are interpreted as the extent to which each marker influences the effect of the other on downstream phenotypes. For example, a negative δ_2 indicates that the presence of marker 2 represses the effect of marker 1 on the phenotypes or eigentraits. The δ terms are related to the main effects and interaction effects as follows:

$$\begin{bmatrix} \beta_1^1 & \beta_2^1 \\ \beta_1^2 & \beta_2^2 \\ \vdots & \vdots \end{bmatrix} \cdot \begin{bmatrix} \delta_1 \\ \delta_2 \end{bmatrix} = \begin{bmatrix} \beta_{12}^1 \\ \beta_{12}^2 \\ \vdots \end{bmatrix} \tag{1}$$

In multiplying out this equation, it can be seen how the δ terms influence each main effect term to give rise to the interaction terms independent of phenotype.

$$\beta_1^j \delta_1 + \beta_2^j \delta_2 = \beta_{12}^j \tag{2}$$

If $\delta_1 = 0$ and $\delta_2 = 0$, there are no addition effects exerted by the markers when both are present. Substitution into the equations above shows that the interaction terms β_{12}^j are 0 and thus the interaction terms have no effect on the phenotype.

Alternatively, consider the situation when $\delta_1 = 1$ and $\delta_2 = 0$. The positive δ_1 indicates that marker 1 should exert an additional effect when marker 2 is present. This can be seen again through substitution into equation 2:

$$\beta_j^1 = \beta_{12}^j$$

These non-zero terms show that there is an interaction effect between marker 1 and marker 2. The positive δ_1 indicates that this interaction is driven through an enhanced effect of marker 1 in the presence of marker 2.

The δs are calculated by solving for equation 1 using matrix inversion:

$$\begin{bmatrix} \delta_1 \\ \delta_2 \end{bmatrix} = \begin{bmatrix} \beta_1^1 & \beta_2^1 \\ \beta_1^2 & \beta_2^2 \\ \vdots & \vdots \end{bmatrix}^{-1} \cdot \begin{bmatrix} \beta_{12}^1 \\ \beta_{12}^2 \\ \vdots \end{bmatrix}$$

This inversion is exact for two eigentraits, and `cape` implements pseudo-inversion for up to 12 eigentraits.

The δ terms are then translated into directed variables defining the marker-to-marker influences m_{12} and m_{21}. Whereas δ_2 described the change in activity of marker 2 in the presence of marker 1, m_{12} can be thought of as the direct influence of marker 2 on marker 1, with negative values indicating repression and positive values indicating enhancement. The terms m_{12} and m_{21} are self-consistent and defined in terms of δ_1 and δ_2:

$$\delta_1 = m_{12}(1 + \delta_2), \ \delta_2 = m_{21}(1 + \delta_1)$$

Rearranging these equations yields the solutions:

$$m_{12} = \frac{\delta_1}{1 + \delta_2}, \ m_{21} = \frac{\delta_2}{1 + \delta_1}.$$

These directed influence variables provide a map of how each marker influences each other marker independent of phenotype. The significance of these influences is determined through standard error analysis on the regression parameters (Bevington, 1994; Carter et al., 2012). This step is particularly important as matrix inversion can lead to large values but larger standard errors, yielding insignificant results. As an example, the variance of m_{12} is calculated by differentiating with respect to all model parameters:

$$\sigma^2_{m_{12}} \cong \sum_{ij} \sigma^2_{\beta^j_i} \left(\frac{\partial m_{12}}{\partial \beta^j_i} \right)^2 + 2 \sum_{i<k, j<l} \sigma^2_{\beta^j_i \beta^l_k} \left(\frac{\partial m_{12}}{\partial \beta^j_i} \right) \left(\frac{\partial m_{12}}{\partial \beta^l_k} \right)$$

In this equation, the indices i and k run over regression parameters and j and l run from 1 to the number of traits. The calculations of the δ and the m terms, as well as the error propagation are performed by the function `error.prop()`.

```
obesity.cross <- error.prop(obesity.cross, perm = FALSE, verbose = FALSE)
```

This function is applied to both the results from the pairwise scan, as well as the permutations of the pairwise scan for later calculation of p values. To apply the calculations to the pairwise scan permutations, set `perm = TRUE`.

```
obesity.cross <- error.prop(obesity.cross, perm = TRUE, verbose = FALSE)
```

For large scans it might be desirable to observe the progress of the calculations. This can be done by setting `verbose = TRUE`. After these calculations have been performed the results of the permutation testing can be used to calculate empirical p values for each of the variant-to-variant effects.

```
obesity.cross <- calc.p(obesity.cross, pval.correction = "fdr")
```

This function also adjusts the empirical p values for multiple testing. The default correction is Holm's stepdown procedure (Holm, 1979). Two other methods, false discovery rate (FDR) (Benjamini and Hochberg, 1995) and local false discovery rate (lFDR) (Liao et al., 2004) are also available. The latter methods of correction for multiple testing are less stringent than the Holm's step-down procedure. The function `calc.p()` adds two elements to the data object. The purpose and returned results of each function is summarized below. For tables of all functions, see the Tables of Functions section at end of this document.

The final step in calculating the network of directed influences is to translate the effect of each marker on the eigentraits to effects on the original phenotypes. The effects of variants on eigentraits are defined in terms of the interaction coefficients m_{12} and m_{21} as well as the main effect of each variant β^j_i. To translate these effects to be in terms of the original phenotypes, the coefficient matrices are multiplied by the singular value matrices $V \cdot W^T$. With two phenotypes and two eigentraits this conversion results in no loss of information. It should also be noted that the translation back to phenotype space does not affect the variant-to-variant influences.

The translation to phenotype space is performed by the function `direct.influence()`.

Functions for Parsing Pair Scan Results

Function	Behavior	Result Label	Structure	Columns
error.prop	propagates errors of coefficients calculated in the pair scan	var.to.var.influences or var.to.var.influences.perm	matrix	marker1, marker2, m12, m21, m12 σ, m21 σ
calc.p	calculates empirical and corrected p values or fdr of interactions using permutations	empirical.p	list of 2 matrices (m12, m21)	marker1, marker2, source, target, influence coefficient, influence σ, empirical p value, adjusted p value or fdr

```
obesity.cross <- direct.influence(obesity.cross, pval.correction = "fdr")
```

In these calculations each marker is assigned multiple direct influences on each phenotype dependent on the marker it was paired with. To reduce the results to a single direct influence of each marker on each phenotype, `direct.influence()` selects the maximum influence observed for each marker. These effects are stored in `max.var.to.pheno.influence`. All effects are accessible in the element `variant.to.phenotype.influences`. To save memory, data from the permutations are not saved by default; however, if `save.permutations` is set to `TRUE` all data are saved in the object `permutation.data.RData`. For details on the elements added to `obesity.cross` and those saved in `permutation.data.RData`, see the Tables of Functions section at end of this document.

The function `direct.influence()` also performs correction for multiple testing for each of the influences. Holm step-down correction (Holm, 1979), FDR Benjamini and Hochberg (1995), and local FDR (Liao et al., 2004) are all available as correction methods.

The marker-to-marker and marker-to-phenotype influences can be plotted with the function `plotVariantInfluences()`. This function plots the adjacency matrix of the final network. It shows all significant influences between variants and between variants and phenotypes.

```
plotVariantInfluences(obesity.cross, p.or.q = 0.01,
all.markers = FALSE, standardize = FALSE, not.tested.col = "lightgray")
```

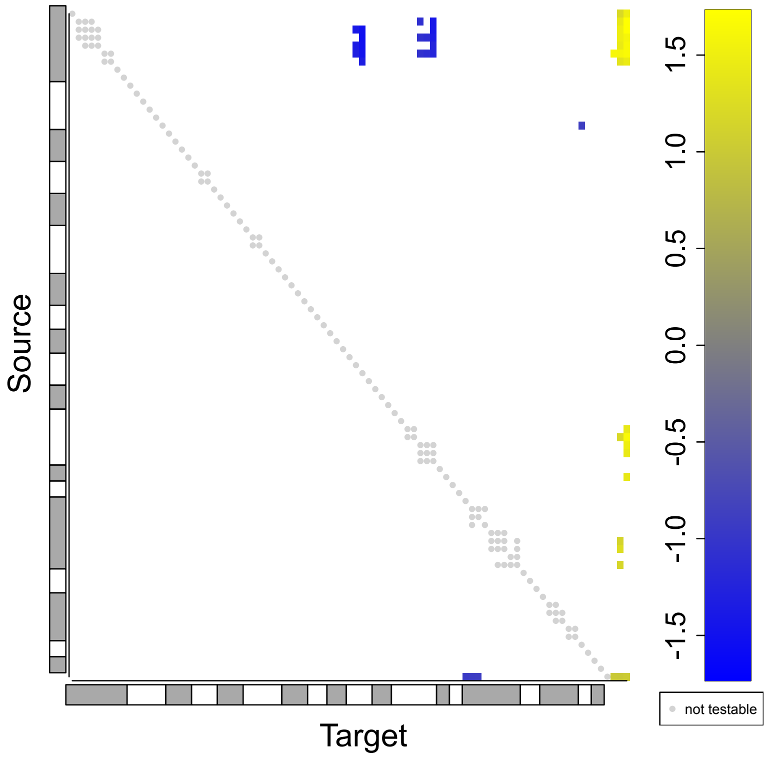

There are several arguments in this function to note. The argument `p.or.q` takes in the p, q, or local fdr value at which interactions are considered significant. Only significant interactions are plotted. Non-significant interactions are colored white. It should be noted that some of the white boxes have gray dots in them. These dots indicate that these pairs were not tested for interactions because they were filtered out due to insufficient representation of individual phenotypes. This usually occurs because the markers are linked and there is low recombination between them. These pairs are marked with dots to distinguish them from pairs that were tested but did not have significant interactions between them. The color of the dots can be changed with the argument `not.tested.col`. The default is `lightgray`. This can be changed to `FALSE` or `NA` if no distinction between not-tested and not-significant is desired. Gray and white bars along the margins of the plot indicate the boundaries of the chromosomes.

It should also be noted that not all of the original markers from the single-locus scan are represented in the final figure. This figure is showing only those markers that were included in the pair scan. The figure can be changed to show all markers by setting `all.markers` to TRUE. However, with large numbers of markers, especially if not all markers are included in the pair scan, the figure can get quite crowded if all markers are included. Finally, the argument `standardize` determines whether the function plots the raw direct influence coefficients or the coefficients divided by standard error.

The table of significant influences can be written to a file using the function `writeVariantInfluences()`.

```
writeVariantInfluences(obesity.cross, p.or.q = 0.05,
filename =  "Significant.Influences.txt")
```

Interpretation of Results

The table of variant-to-variant and variant-to-phenotype influences is the primary output of `cape`. The function for plotting results, `plotVariantInfluences()`, plots an asymmetric adjacency matrix, which is useful for identifying patterns in variant interactions.

Another useful visualization of the network can be plotted using `plotNetwork()`. This function plots the chromosomes linearly and shows interactions as arrows between locations on chromosomes (see figures below). Main effects are plotted beneath the chromosomes. Before plotting the network in this format, the function `get.network()` must be be run to create a network object. The function `get.network()` network offers the option of condensing markers based on their linkage. The minimum Pearson R^2 threshold separating two markers is provided in the argument `r2.thresh`. The argument `p.or.q` determines the p, q, or local FDR value at which marker influences are determined significant.

```
obesity.cross <- get.network(obesity.cross, p.or.q = 0.01,
collapse.linked.markers = TRUE, r2.thresh = 0.8, standardize = FALSE)
plotNetwork(obesity.cross, collapsed.net = TRUE)
```

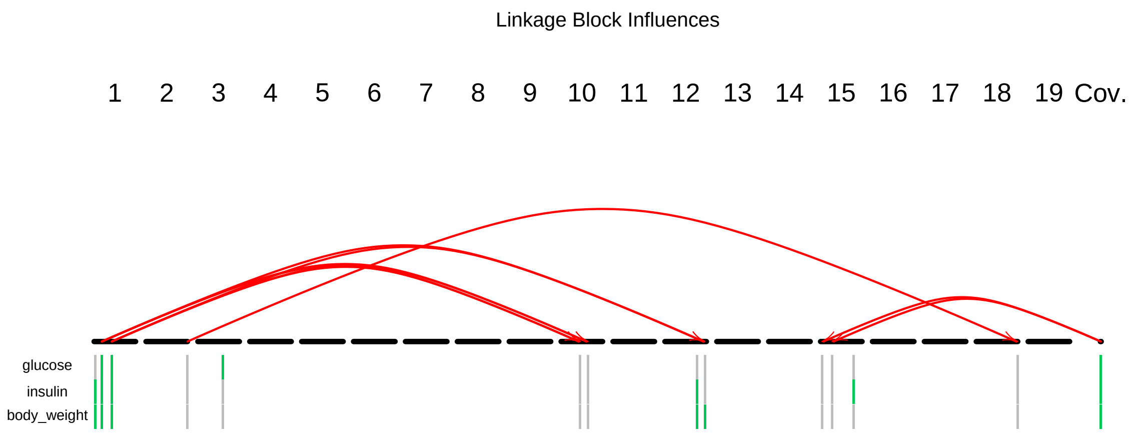

If no collapse by linkage is desired, the argument `collapse.linked.markers` can be set to `FALSE` and `r2.thresh` will be ignored.

```
obesity.cross <- get.network(obesity.cross, p.or.q = 0.01,
collapse.linked.markers = FALSE, standardize = FALSE)
plotNetwork(obesity.cross, collapsed.net = FALSE)
```

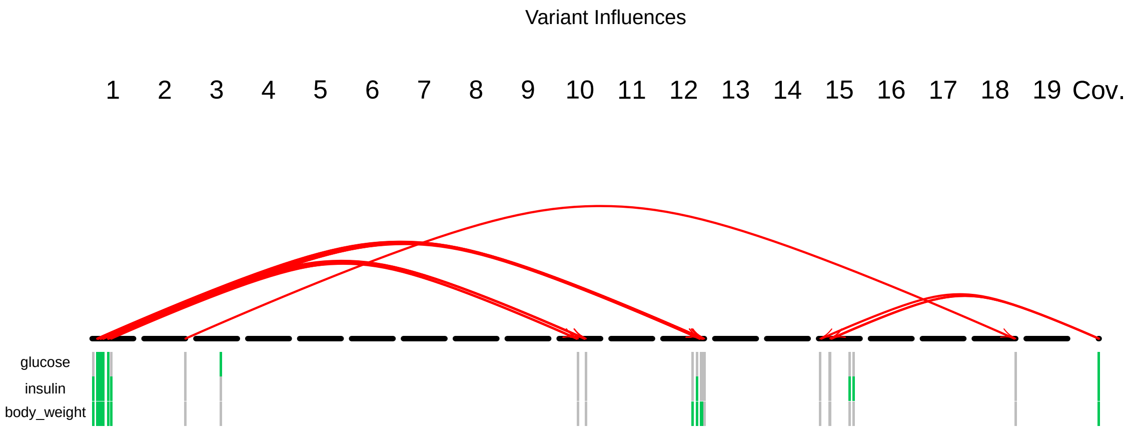

Both the network figure and the adjacency matrix show direct influences of markers on the phenotypes as well as interactions between markers. As expected, NZO variants on multiple chromosomes show positive effects on plasma insulin and glucose levels as well as on body weight. Maternal obesity was found to suppress the effects of markers on Chromosome 15, and a marker on Chromosome 2 suppressed the effects of a marker on Chromosome 18. In the latter interaction, neither marker had an individual main effect, although the Chromosome 18 marker has a marginally significant effect ($q = 0.018$) on body weight that is completely suppressed by the Chromosome 1 marker. NZO variants in a region on Chromosome 1 increased all three phenotypes individually and were the hub of a small epistatic network suppressing the effects of markers on Chromosomes 10 and 12.

The interaction between Chromosomes 1 and 12 is illustrative of the R/cape strategy. Although both loci have a significant effect on body weight, their joint effect is less than additive (See figure below). This finding suggests one locus might be suppressing the effect of the other, but is ambiguous about the potential direction of that suppression. In contrast, only the Chromosome 1 locus has an effect on insulin and this effect is independent of the

Chromosome 18 locus. This second phenotype provides R/cape the information necessary to infer the directionality of the interaction from Chromosome 1 to 12, since a reversed interaction would imply epistasis for insulin along with body weight, which was not observed. R/cape thus provides a more stringent hypothesis for gene candidates in the two loci through the constraint of directional genetic effects.

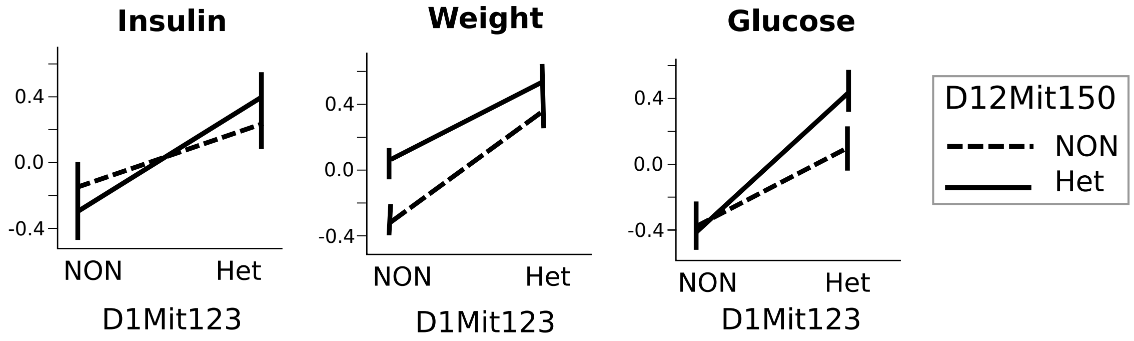

These findings illustrate how `cape` is designed to find interactions that simultaneously model all phenotypes under the assumption that interactions between variants across multiple contexts represent a single underlying interaction network. Thus we recommend users assess single-phenotype epistasis using functions in `cape` or in parallel analyses using tools such as `R/qtl` and `R/qtlbim` (Yandell et al., 2007).

Tables of Functions

Basic `cape` Functions

Reading and Writing Data	
read.population	read in population data
writeVariantInfluences	write out a table of the variant influences to each other and the variant influences on traits.

Data Manipulation	
create.covar	assign a phenotypic value as a covariate in the genotype matrix
delete.pheno	delete the specified phenotypes from the phenotype matrix
get.covar	automatically select covariates for the pairwise scan based on a user-defined threshold of β/σ
norm.pheno	use rank Z normalization to normalize the phenotypes
select.eigentraits	select a subset of eigentraits to use in the analysis
select.pheno	select a subset of phenotypes to use in the analysis
select.by.chr	select a subset of chromosomes to use in the analysis
select.by.ind	subset the individuals in the population by either phenotypic or genotypic values
set.covar	manually set markers to be used as covariates for individual traits
set.pairscan.thresh	manually set the threshold for inclusion of markers in the pairscan

Plotting Functions	
plotNetwork	plot a network view of the significant influences of variants on each other and variants on traits
plotSinglescan	plot the results of the single-locus scan or markers selected for pairscan
plotPairscan	plot the results of the pair scan
plotSVD	plot the results of the singular value decomposition
plotVariantInfluences	plot the adjacency matrix showing the significant influences of variants on each other and variants on traits

Functions for cape Analysis

Function	Behavior	Result Label	Structure	Internal elements	Each Row	Columns
calc.p	calculates empirical and corrected p values of interactions using permutations	empirical.p	list of 2 matrices	m12, m21	marker pair	marker1, marker2, source, target, influence coefficient, influence σ, empirical p value, adjusted p value
direct.influence	calculates the direct influence of each marker pair on each trait and the p value of each influence	var.to.pheno.influence	list of N_t* matrices	each matrix is named with its respective trait	marker pair	marker1, marker2, influence coefficients and standard errors for each marker on each trait
		var.to.pheno.influence.perm†	list of N_t* matrices	each matrix is named with its respective trait	permutation of each marker pair	marker1, marker2, permuted influence coefficients and standard errors for each marker on each trait
		var.to.pheno.test.stat†	list of N_t* matrices	each matrix is named with its respective trait	one marker in one marker pair context	marker, influence coefficient, σ, t statistic, |t statistic|
		var.to.pheno.test.stat.perm†	list of N_t* matrices	each matrix is named with its respective trait	one permutation for each marker in one marker pair context	marker, influence coefficient, σ, t statistic, |t statistic|
		max.var.to.pheno.influence	list of N_t* matrices	each matrix is named with its respective trait	marker	marker, influence coefficient, σ, t statistic, |t statistic|, empirical p value, adjusted p value
error.prop	propagates errors of coefficients calculated in the pair scan	var.to.var.influences or var.to.var.influences.perm	matrix		marker pair	marker1, marker2, m12, m21, m12 σ, m21 σ
get.eigentraits	performs singular value decomposition on phenotype matrix	ET	matrix		individual	one column for each eigentrait
maximum.influence	calculates the maximum influence that each marker has on each trait across all pair contexts	max.var.to.pheno.influence	list of N_t* matrices	each matrix is named with its respective trait	marker pair	marker, influence coefficient, σ, t statistic, |t statistic|, empirical p value, adjusted p value
singlescan	performs all single-locus regressions for each marker on each trait	singlescan.results	list of N_t* matrices	each matrix is named with its respective trait	marker	marker coefficient, σ, t statistic, p value
pairscan(n.perm $\geq$ 0)	performs pair-wise marker regression for each trait	pairscan.results	list of N_t* lists, each with 3 elements	twoD.effects	marker pair	marker labels and coefficients for each marker tested and each marker used as a covariate, including the interaction term
				twoD.se	marker pair	marker labels and σ for each coefficient in twoD.effects
				model.covariance	marker pair	the model covariance matrix for each regression
pairscan(n.perm $\geq$ 1)	performs pair-wise marker regression and permutations for each trait	pairscan.perm	list of N_t* lists, each with 3 elements	twoD.effects.perm	one permutation of marker pair	marker labels and coefficients for each marker tested and each marker used as a covariate, including the interaction term for all permutations
				twoD.se.perm	one permutation of marker pair	marker labels and σ for each coefficient in twoD.effects.perm
				model.covariance.perm	one permutation of marker pair	the model covariance matrix for each permuted regression
select.markers.for.pairscan	selects markers for the pair scan based on which markers are linearly independent of the other markers in the data set	geno.for.pairscan	matrix		individual	one column for each independent marker selected for the pair scan
		covar.for.pairscan	matrix		marker	each of the N_t* columns indicates which markers are to be used as covariates in the pair scan of each trait

* N_t = Number of traits (phenotypes or eigentraits) scanned

† These objects are not added to the data object, but saved in `permutation.data.RData` if specified.

References

Benjamini, Y. and Hochberg, Y. (1995). Controlling the false discovery rate: a practical and powerful approach to multiple testing. *Journal of the Royal Statistical Society. Series B (Methodological)*, pages 289–300.

Bevington, P. (1994). *Data reduction and error analysis for the physical sciences, ise*. McGraw-Hill, New York.

Broman, K. W., Sen, S., Owens, S. E., Manichaikul, A., Southard-Smith, E. M., and Churchill, G. A. (2006). The X chromosome in quantitative trait locus mapping. *Genetics*, 174(4):2151–2158.

Broman, K. W., Wu, H., Sen, S., and Churchill, G. A. (2003). R/qtl: QTL mapping in experimental crosses. *Bioinformatics*, 19:889–890.

Burcelin, R., Crivelli, V., Dacosta, A., Roy-Tirelli, A., and Thorens, B. (2002). Heterogeneous metabolic adaptation of C57BL/6J mice to high-fat diet. *American journal of physiology. Endocrinology and metabolism*, 282(4):E834–42.

Carter, G. W., Hays, M., Sherman, A., and Galitski, T. (2012). Use of pleiotropy to model genetic interactions in a population. *PLoS genetics*, 8(10):e1003010.

Das, S. K. and Elbein, S. C. (2006). The Genetic Basis of Type 2 Diabetes. *Cellscience*, 2(4):100–131.

Haffner, S. (2003). Epidemic Obesity and the Metabolic Syndrome. *Circulation*, 108(13):1541–1545.

Holm, S. (1979). A simple sequentially rejective multiple test procedure. *Scandinavian journal of statistics*, pages 65–70.

Liao, J. G., Lin, Y., Selvanayagam, Z. E., and Shih, W. J. (2004). A mixture model for estimating the local false discovery rate in DNA microarray analysis. *Bioinformatics*, 20(16):2694–2701.

Mackay, T. F. C., Richards, S., Stone, E. A., Barbadilla, A., Ayroles, J. F., Zhu, D., Casillas, S., Han, Y., Magwire, M. M., Cridland, J. M., Richardson, M. F., Anholt, R. R. H., Barrón, M., Bess, C., Blankenburg, K. P., Carbone, M. A., Castellano, D., Chaboub, L., Duncan, L., Harris, Z., Javaid, M., Jayaseelan, J. C., Jhangiani, S. N., Jordan, K. W., Lara, F., Lawrence, F., Lee, S. L., Librado, P., Linheiro, R. S., Lyman, R. F., Mackey, A. J., Munidasa, M., Muzny, D. M., Nazareth, L., Newsham, I., Perales, L., Pu, L.-L., Qu, C., Ràmia, M., Reid, J. G., Rollmann, S. M., Rozas, J., Saada, N., Turlapati, L., Worley, K. C., Wu, Y.-Q., Yamamoto, A., Zhu, Y., Bergman, C. M., Thornton, K. R., Mittelman, D., and Gibbs, R. A. (2012). The Drosophila melanogaster Genetic Reference Panel. *Nature*, 482(7384):173–178.

Permutt, M. A., Wasson, J., and Cox, N. (2005). Genetic epidemiology of diabetes. *The Journal of clinical investigation*, 115(6):1431–1439.

Reifsnyder, P. C. (2000). Maternal Environment and Genotype Interact to Establish Diabesity in Mice. *Genome Research*, 10(10):1568–1578.

Yandell, B. S., Mehta, T., Banerjee, S., Shriner, D., Venkataraman, R., Moon, J. Y., Neely, W. W., Wu, H., von Smith, R., and Yi, N. (2007). R/qtlbim: QTL with Bayesian Interval Mapping in experimental crosses. *Bioinformatics*, 23(5):641–643.
